# Evaluating the influential priority of the factors on insurance loss of public transit

**DOI:** 10.1371/journal.pone.0190103

**Published:** 2018-01-03

**Authors:** Wenhui Zhang, Yongmin Su, Ruimin Ke, Xinqiang Chen

**Affiliations:** 1 School of Traffic and Transportation, Northeast Forestry University, Harbin, China; 2 Department of Civil and Environmental Engineering, University of Washington, Seattle, United States of America; 3 Merchant Marine College, Shanghai Maritime University, Shanghai, China; Beihang University, CHINA

## Abstract

Understanding correlation between influential factors and insurance losses is beneficial for insurers to accurately price and modify the bonus-malus system. Although there have been a certain number of achievements in insurance losses and claims modeling, limited efforts focus on exploring the relative role of accidents characteristics in insurance losses. The primary objective of this study is to evaluate the influential priority of transit accidents attributes, such as the time, location and type of accidents. Based on the dataset from Washington State Transit Insurance Pool (WSTIP) in USA, we implement several key algorithms to achieve the objectives. First, K-means algorithm contributes to cluster the insurance loss data into 6 intervals; second, Grey Relational Analysis (GCA) model is applied to calculate grey relational grades of the influential factors in each interval; in addition, we implement Naive Bayes model to compute the posterior probability of factors values falling in each interval. The results show that the time, location and type of accidents significantly influence the insurance loss in the first five intervals, but their grey relational grades show no significantly difference. In the last interval which represents the highest insurance loss, the grey relational grade of the time is significant higher than that of the location and type of accidents. For each value of the time and location, the insurance loss most likely falls in the first and second intervals which refers to the lower loss. However, for accidents between buses and non-motorized road users, the probability of insurance loss falling in the interval 6 tends to be highest.

## Introduction

The purpose of vehicle insurances is to cover the claims of policyholders from accidents. Vehicles without insurance are forbidden to run on public roads in many countries. This kind of compulsory regulation ensures reasonable financial compensations for losses of the third parties involved in accidents. Besides the compulsory third-party liability coverage, most insurers offer the first-party coverage aiming to provide compensation for the insured party (vehicle damages and personal injuries).

Currently, most insurers use the performance of vehicles, claim counts and previous insurance losses to calculate the future premium [1–4]. Being an independent variable, the insurance loss is one of the most important statistics, since it has been a basis for complicatedly pricing [5–7]. A great many of bonus-malus systems use the claims number to optimize services and improve competitiveness of insurers as well [8–12].They proposed that not all accidents produced the same individual claim size and thus it did not seem fair to penalize all policyholders in the same way when claims were presented. Therefore, the number of claims should not be the only basis of future premium. The amount of claims and some other factors should be taken into account.

In addition, the insurance loss is directly associated with the profits of an insurance company as well. All the insurers try to gain an equilibrium outcome every year. Consequently, it is of importance to conduct a deep analysis on insurance loss data.

Most of the previous studies about insurance loss focused on data fitting and modeling. As the insurance loss data were characterized by non-negative, asymmetry and heavy tails, researchers applied Erlang [13] and Gamma kernel [14] to fit distribution of the insurance loss. These models explained the loss data very well and provided an accurate estimation for future costs. Gradient boosting trees[15]and [16] A Bayesian non-linear model were applied to forecast the insurance loss and claim amounts. Some researcher assumed that claim frequency and severity were often dependent. The generalized linear model [17, 18] and regression model [19] were applied to describe their relations. Insurers could employ theses estimating models to actuarial decision, e.g. for pricing insurance contracts and for calculation of premium.

However, limited literature concentrated on the correlation between the insurance loss and the accidents characteristics. The heavy tails that insurance loss data exhibited indicated that the insurance losses of a small number of accidents were impressive high [20]. Exploring features of these accidents was significant for both transit companies and insurance companies.

As the most popular travel pattern, the public transit basically runs on the time schedule and planned routes, all the drivers are professional[21–23]. However, the premium and insurance loss tends to be more than that of the private cars. It is crucial for insurance companies and transit companies to find out the key influential factors on insurance losses. A great number of studies have proven that Grey Relational Analysis (GCA) and Naïve Bayes theory are effective for evaluating correlations between influential factors and systems [24–27]. To this extent, we firstly explored the influential priority of factors by use of GRA; then applied Naïve Bayes theory to calculate the probabilities of factor values to insurance loss. This study attempted to provide theoretical evidences for transit insurance companies to adopt the most targeted countermeasures for insurance loss minimization.

This paper is constructed as follows. In section 2, the data source and descriptions are summarized. K-means algorithm used to cluster the insurance loss data, GRA and Naïve Bayes theory used to identify the main influential factors are briefly described in section 3. And section 4 presents the results and discussions. At last, the conclusions and future studies are provided.

## Data source and descriptions

### Data collection and descriptions

The loss data of transit insurance used in this study were collected from the Washington State Transit Insurance Pool (WSTIP) which consisted of 25 Washington public transit agencies. The dataset included the number, time, location, detailed description, transit route and insurance loss of every claim. The insurance loss covered the cost associated with the bodily injuries and property damages of the third party. There were a total of 4990 available cases recorded from January 1, 2004 to March 31, 2016. Table 1 illustrated the primary statistics of the loss data of transit insurance.

**Table 1:** Statistics summaries for loss data of transit insurance.

Statistic	Sample size	Max ($)	Min ($)	Mean ($)	Std. dev. ($)	Skewness	Excess kurtosis
Value	4.99e3	3.58e6	7	1.30e4	1.05e5	21	557

We divided the gross loss data into 11 sections at intervals of 5000. The proportions of the insurance loss and the number of accidents in each interval were shown in S1 Fig.

It could be concluded from S1 Fig that the number of accidents lower than $5000 accounted for 78.33%, but the proportion of insurance loss was only 7.63%. However, the number of accidents more than $25,000 only accounted for 6.65%, the proportion of insurance loss was impressively as high as 79.28%. A small number of accidents accounted for the majority of the gross insurance loss. This finding probably provided a good evidence for insurers and policyholders to realize the importance of reducing these accidents.

In order to observe the distribution of accident losses, we applied the corresponding frequency distribution histogram, as shown in S2 Fig. The horizontal axis of histogram showed data packets and the vertical axis showed frequency, and probability density function could express this distribution. It could be found that log-logistic distribution fitted the best, and the value of Kolmogorov Smirnov (*K-S*) test and Anderson Darling (*A-D*) test was *P*_*K*−*S*_ = 0.16 and *P*_*A*−*D*_ = 0.25 respectively.

### Influential factors

Based on the dataset, the most relevant explanatory factors were the time, location and accident type. The time of accidents was classified into three categories which were peak time, day time and night time. The location of accidents was classified into five categories which were street, intersection, roadway, not departure and inside transit. The accidents type was classified into three categories which were the collisions between buses and non-motorized, bus and motorized, other types. Table 2 showed the detailed information of three factors.

**Table 2:** Variables descriptions of insurance loss data.

Factors	Variables and Descriptions	Percentage (%)
Time	1 Peak time	20.18
	7:00~9:00 and 16:00~18:00	
	2 Day time	39.34
	9:00~16:00	
	3 Night time	40.48
	18:00~7:00	
Location	1 Street	42.18
	Street, crosswalk, walkway, alley	
	2 Intersection	21.02
	3 Roadway	13.12
	Freeway, highway, rural road	
	4 Not departure	14.07
	Shopping center/mall, parking lot/facility, transit center	
	5 Inside transit	9.52
Type	1 Bus with non-motorized	8.08
	Bus with pedestrian and bicyclists	
	2 Bus with motorized	65.51
	Bus with car, bus, truck, van	
	3 Others	26.41
	Inside transit, bus with other infrastructures	

Box-plot was employed to reveal the relationships between each factor and insurance loss, as shown in S3–S5 Figs. It could be concluded that the accidents occurring in night time tended to cause higher insurance loss. If accidents occurred on roadways and intersections, the insurance loss tended to be higher. The insurance loss was higher if buses collided with the non-motorized road users. However, the influential priority of three factors could not be found from S3–S5 Figs.

## Methodology

Since the range of insurance loss data was wide, from $ 6.75 to $ 3,575,000, it was advisable to divide the loss data into several intervals. If the interval of loss was equal, the first interval tended to consist of majority of data. We used K-means algorithm to cluster the insurance loss data, then GRA was applied to calculate the influential priority of three factors, Bayes theory was applied to explore the probability of factor values to each interval of loss. These models were described in the following section.

### K-means

*K*-Means is one of the efficient algorithms to address a clustering problem by use of a simple iterative scheme for searching a locally minimal solution. The basic objective of this algorithm is to divide a dataset samples into *K* groups with the maximum inter-cluster distances and the minimum intra-cluster distances. Each cluster has a centroid located in a problem space. The investigators therefore obtain the reasonably similar groups from *N*-dimensional data. The method is applicable for a close interaction with theory and intuition.

*C*_*i*_(*i* = 1,2,⋯,*K*) are defined as the current K centroids which are randomly chose. The first step is to calculate the distance between each object to the initial center point and associate each other based on nearest distance. Next to recalculate the new *K* center points according to the previous step. These two calculating procedures are iteratively repeated until convergence to get the optimum assignments for each center point. The Euclidean distance is mostly used to calculate the distance between each object to the center point. The basic optimum function is shown as follows:

$$minf=\sum_{i=1}^{K}\sum_{j=1}^{N}\left\|x_{j}-C_{i}\right\|\;j\in G_{i}$$ (1)

Where *K* is defined as the number of clusters, *N* is the number of objects, *C*_*i*_ is the coordinate of the centroid in cluster *i*, *G*_*i*_ is the objects group belonging to the cluster *i*.

To minimize the intra-cluster distances, the center points should be adjusted by averaging the location of all objects assigned to it, as shown in Eq (2):

$$C_{i}=\frac{1}{N}\sum_{j=1}^{N}x_{j}\;j\in G_{i}$$ (2)

### Grey Relational Analysis

GRA is an effective algorithm for evaluating the relationship between the data sequences which include the compared series and the reference series. This method is based on the calculation of the Grey Relational Grades (GRGs) to evaluate the level of correlation. The higher is the GRG value, the better is the corresponding multiple performance characteristic. In this paper, three factors (time, location and accident type) were taken as the compared series and the insurance loss was used as the reference series. The process of GRA includes three steps: grey relational generating, grey relational coefficient calculating and grey relational grade calculating.

As different factors usually have different units, it is necessary to normalize the original sequence into [0,1]. If the number of the factors is m and the number of attributes is n, the compared and reference series can be express as *Y*_*j*_ = (*y*_*ij*_) (*i* = 1,2,⋯,*m*; *j* = 1,2,⋯,*n*) and *Y*_0_ = (*y*_0*j*_) (*j* = 1,2,⋯,*n*) respectively. *Y*_*i*_(*i* = 0,1,⋯,*m*) can be translated into the comparability sequences *X*_*i*_ = (*x*_*ij*_) (*i* = 0,1,⋯,*m*; *j* = 1,2,⋯,*n*) by means of the following three equations.

$$x_{ij}=\frac{y_{ij}-min\{y_{ij}\}}{max\{y_{ij}\}-min\{y_{ij}\}}(i=0,1,\cdots ,m;j=1,2,\cdots ,n)$$ (3)

$$x_{ij}=\frac{max\{y_{ij}\}-y_{ij}}{max\{y_{ij}\}-min\{y_{ij}\}}(i=0,1,\cdots ,m;j=1,2,\cdots ,n)$$ (4)

$$x_{ij}=1-\frac{\left|y_{ij}-y_{j}^{*}\right|}{max\{max\{y_{ij}\}-y_{j}^{*},y_{j}^{*}-min\{y_{ij}\}\}}(i=0,1,\cdots ,m;j=1,2,\cdots ,n)$$ (5)

Eq (3) is applied for the larger-the better attributes, Eq (4) is applied for the smaller-the better attributes and Eq (5) is applied for the closer to the desired value $y_{j}^{*}$-the better attributes.

According to the grey relational sequence, the grey relational coefficient is calculated to reveal the relationship between the compared and the reference series. The grey relational coefficient can be calculated by Eq (6).

$$\gamma (x_{0j},x_{ij})=\frac{{∆}_{min}+\rho {∆}_{max}}{{∆}_{ij}+\rho {∆}_{max}}(i=1,2,\cdots ,m;j=1,2,\cdots ,n)$$ (6)

where *γ* is the grey relational coefficient and Δ_*ij*_ = |*x*_0*j*_ − *x*_*ij*_|, Δ_*min*_ = *min*{Δ_*ij*_}Δ_*max*_ = *max*{Δ_*ij*_} *(i* = 1,2,⋯,*m*; *j* = 1,2,⋯,*n)*, *ρ (*0 < *ρ* ≤ 1*)* is a distinguishing coefficient which is usually taken as 0.5 in most studies.

A grey relational grade can be calculated by use of the average value of grey relational coefficients, which reveals the influential priority of factors on the reference series. The grey relational grade can be computed by Eq (7).

$$\tau (Y_{0},Y_{j})=\frac{1}{n}\sum_{j=1}^{n}\gamma (x_{0j},x_{ij})(i=1,2,\cdots ,m;j=1,2,\cdots ,n)$$ (7)

### Naïve Bayes algorithm

Naïve Bayes algorithm is usually used to estimate the probability of an observation belonging to a predefined category. This learning method is based on the observed data to calculate the prior probability. Then the posteriori probability can be assessed by use of a conditional probability function. Despite its independence assumption, Bayes algorithm is probed to be quite useful in modeling the condition of complex real-world problems and is widely used for decision making and inferential analysis.

The equation of posteriori probability is shown as follows:

$$P(Y_{i}/X)=\frac{P(X/Y_{i})∙P(Y_{i})}{P(X)}$$ (8)

Where *P*(*Y*_*i*_/*X*) is the posteriori probability which means the probability of the observed variable X belonging to category *Y*_*i*_, *P*(*X*/*Y*_*i*_) is the probability of *X* given category *Y*_*i*_, *P*(*Y*_*i*_) is the prior probability of category *Y*_*i*_, and *P*(*X*) is the prior probability of *X* according to the training data.

Naïve Bayes algorithm takes advantage of the maximum likelihood to classify multiple variables based on Bayes theory. The algorithm holds that the effect of a variable on a given category is independent of the other variables. Normally, if (*Y*_*i*_/*X*) > *P*(*Y*_*j*_/*X*)(*i* ≠ *j*), the algorithm assumes that X belongs to *Y*_*i*_, which is the theory of Naïve Bayes classifier. This paper used the Naïve Bayes to calculate the probability of the variables belonging to the loss category.

## Results

### Interval division of loss

In order to observe the influential priority of three factors on the loss in-depth, the gross loss data was divided into some small intervals in which the grey relational grades were calculated respectively. S1 Fig had shown that the sample size over $25000 was 334 and the loss in this interval account for 79.28% of the total. Accordingly, the data over 25000 was classified as one category.

*K*-means algorithm was performed to cluster the gross loss lower than $ 25000 into five sections. According to Eq 1 and Eq 2, the clustering results were shown in Table 3 and S4 Fig.

**Table 3:** *K*-means cluster and statistics of insurance loss data.

Statistic	Loss section					
	1	2	3	4	5	6
Sample size	2.82e3	1.04e3	406	245	141	334
Max	1.71e3	4.67e3	9.61e3	1.65e4	2.48e4	3.58e6
Min	7	1.71e3	4.67e3	9.66e3	1.65e4	2.50e4
Mean	656	2.77e3	6.61e3	1.27e4	2.06e4	1.55e5

### Relational grade calculation

GRA was used to calculate the influential priority of the time, location and accident type on the loss of transit insurance. Firstly, all the loss data were normalized into [0,1]. Then the grey relational coefficients of insurance loss were computed using Eq (6). The grey relational grades of three factors were calculated using Eq (7). Table 4 showed the grey relational grades of three factors to gross loss and each interval.

**Table 4:** Grey relational grades of factors to insurance loss.

Factors	Gross Loss	Loss Interval					
		1	2	3	4	5	6
Time	0.9859	0.6836	0.6406	0.7109	0.7500	0.4905	0.9139
Location	0.0078	0.7426	0.6911	0.6969	0.6567	0.5588	0.0553
Type	0.0063	0.6802	0.6263	0.6677	0.7790	0.4963	0.0554

The maximum value of the grey relational grades was 0.9859 which showed significantly higher, followed by the location. The grey relational grade of the type was smallest. It indicated that the influential priority of the three factors on gross insurance loss was time, location and type. However, in section 1 to 5, all the factors had significant influence on the insurance loss and the grey relational grades had no significant difference. In the section 6, however, the time of accident became the predominant influential factor, followed by accident type and location. The results filled the gap of S3–S5 Figs by evaluating the influential priority of three factors.

Actually, based on these findings the insurers could modify their policy according to the different time, location and type of accidents. As time tended to be the most concerned factor, the insurers may modify policy-holders’ premium on basis of claims number during given hours. For example, if claims number of a policy-holder at night time is higher than the limit, the insurers may increase the premium next year. In addition, the transit companies could pay more attention to some routes and take more targeted countermeasures on the crucial hour to reduce the number of accidents[28]. Nevertheless, the relationships between values of each factor and loss intervals were unable to be evaluated only by GRA. Thus the key time, location and type of accidents should be further identified.

### Inferential analysis for insurance loss

Naïve Bayes theory was used to calculate the inference probability between each value of factors and loss intervals of transit insurance. The insurance loss data was used as training set to calculate the prior probability. The posteriori probabilities of each value of factors to loss intervals were calculated according to Eq 3, as shown in Table 5.

**Table 5:** Posteriori probability of factors values to loss intervals.

Factor	Value	Insurance Loss Interval					
		1	2	3	4	5	6
Time	1	0.6934	0.2386	0.0495	0.0099	0.0039	0.0049
	2	0.5181	0.1544	0.1004	0.0754	0.0388	0.1013
	3	0.4002	0.2572	0.1082	0.0765	0.0576	0.1131
Location	1	0.6095	0.2138	0.0651	0.0356	0.0209	0.0551
	2	0.4423	0.2526	0.1163	0.0715	0.0391	0.0781
	3	0.4006	0.1821	0.1381	0.0926	0.0577	0.1290
	4	0.6923	0.2066	0.0399	0.0199	0.0114	0.0299
	5	0.6779	0.1347	0.0589	0.0421	0.0232	0.0631
Type	1	0.2333	0.1042	0.0471	0.0794	0.1886	0.3474
	2	0.5552	0.2463	0.0918	0.0511	0.0144	0.0413
	3	0.6904	0.1495	0.0661	0.1017	0.0144	0.0448

In terms of the time of accidents, no matter what time the accidents occurred, in the peak time, day time or night time, for example, the probabilities of the insurance loss falling in the intervals 1 and 2 were higher, added up to more than 65%, being consistent with the results in S1 Fig. The accidents in day time and night time caused higher probability of the insurance loss falling in interval 6 than that in peak time (*P*(*I* = 6/*T* = 1) = 0.0049, *P*(*I* = 6/*T* = 2) = 01013, *P*(*I* = 6/*T* = 3) = 0.1131). The results agreed with the previous achievements [29, 30]. For instance, Shumin Feng et al. (2016) found that driving buses in evening and night more likely caused severe accidents compared to accidents occurring in the morning. For other road accidents, Akerstedt et al. (2001) found that the highest total accident risk was seen at 04:00 h and fatal accidents most likely occurred at this point. Insurers may implement dynamic pricing that means increasing the premium rate of accidents occurring in night, or decrease the premium for buses involving less number of accidents occurring in night. Transit agencies may modify the reasonable final bus hour or consider the rationality of driver substitution according our findings.

In terms of the location of accidents, no matter where accidents occurred, street, roadway, parking et al. for example, the probabilities of insurance loss falling in 1 and 2 section were higher, added up to more than 60%. The accidents occurring in roadway (location 3, freeway, highway and rural way) resulted in the highest probability of insurance loss falling in interval 6 (*P*(*I* = 6/*L* = 3) = 0.1290), followed by intersection (location 2) and inside transit (location 5) (*P*(*I* = 6/*L* = 2) = 0.07814, *P*(*I* = 6/*L* = 5) = 0.0631). When the buses run in freeway, highway or rural way, their speed tend to be higher. Once accidents occur, the casualties are severe. This result agrees with the previous study [31] that the higher speed is, the more severe accidents tend to be. These findings provided good evidences for the insurers to modify their bonus-malus system for future premium. For instance, for the policyholders involved in less in less accidents occurring on roadway, insurers may reduce their future premiums, otherwise, increase their premiums. Transit agencies may take more targeted countermeasures to limit the speed of buses to reduce the number of some severe accidents.

In terms of the type of accidents, when collisions between buses and pedestrian or bicycles occurred, the probability of insurance loss falling in interval 6 was found to be highest (*P*(*I* = 6/*Ty* = 1) = 0.3474), followed by type 1 and 5. It indicated that accidents involved non-motorized road users either caused the severe casualties or caused minor injuries. Compared with buses, pedestrian or bicycles had lighter weight. Once buses impacted them, the injuries tended to be severe which caused the higher insurance loss. When collisions between buses and vehicles and other type of accidents occurred, the probabilities of insurance loss falling in intervals 1 were higher, added up to more than 80%. Insurers should pay more attention to accidents involved pedestrian and bicycles that more likely result in high insurance loss. And the targeted bonus-malus system should be considered to perform.

## Conclusions

The insurance loss has direct relationship with profits of an insurer. Understanding the effects of influential factors on insurance loss may help develop targeted countermeasures. Although there have been many achievements on loss fitting models, limited researches were conducted to explore the interaction between losses and the time, location and type of transit accidents. Based on 4990 insurance loss records from WSTIP, this study took the time, location and type of accidents into account and applied GRA and Naïve Bayes to explore the influence priority of factors.

Although the number of accidents with insurance loss more than $ 25,000 was small, the insurance losses accounted for about 80% of total losses. Insurers should pay more attention to these accidents and try to reduce the number of these accidents. K-means algorithm clustered the gross insurance data in 6 intervals. In the first five intervals, the time, location and type of accidents significantly influenced insurance losses. In the interval 6, where the insurance loss was higher, the time of accidents was a significant influential factor.

No matter the accidents occurred in peak time, day time or night time, they tended to cause the lower insurance loss. And no matter the accidents occurred in streets, intersections, roadways, parking or inside transit, the probabilities of insurance loss falling in intervals 1 and 2 were higher. However, collisions between buses and non-motorized road users tended to cause the more insurance loss than other types of accidents. These findings were beneficial for both insurers and transit companies. The insurers may increase or decrease premium on basis of the claims performance during previous year. The companies may evaluate the drivers’ performance with consideration of their claims as well.

These findings may help the insurers better understand the most influential factors, and then try to take targeted countermeasures to reduce the losses. Although Naïve Bayes can help us calculate the posterior probability of influential factors to loss, this method assumes that the influence of each factor on losses is independent. The Bayesian Network has been used to address the correlation between factors in recent years [32–34],further researches can be conducted based on this method.

## Supporting information

S1 Fig Proportions of the insurance loss and the accidents number. (TIF)

S2 Fig Fitting distribution of insurance loss. (TIF)

S3 Fig Box-plots of factors and insurance loss (Time). (TIF)

S4 Fig Box-plots of factors and insurance loss (Location). (TIF)

S5 Fig Box-plots of factors and insurance loss (Accident type). (TIF)

S6 Fig K-means cluster of insurance loss data. (TIF)
