# Supplementary figures and images for: Evaluating the influential priority of the factors on insurance loss of public transit
Source: PLoS One. 2018 Jan 3;13(1):e0190103. doi: 10.1371/journal.pone.0190103 (PMC5752032; doi:10.1371/journal.pone.0190103)

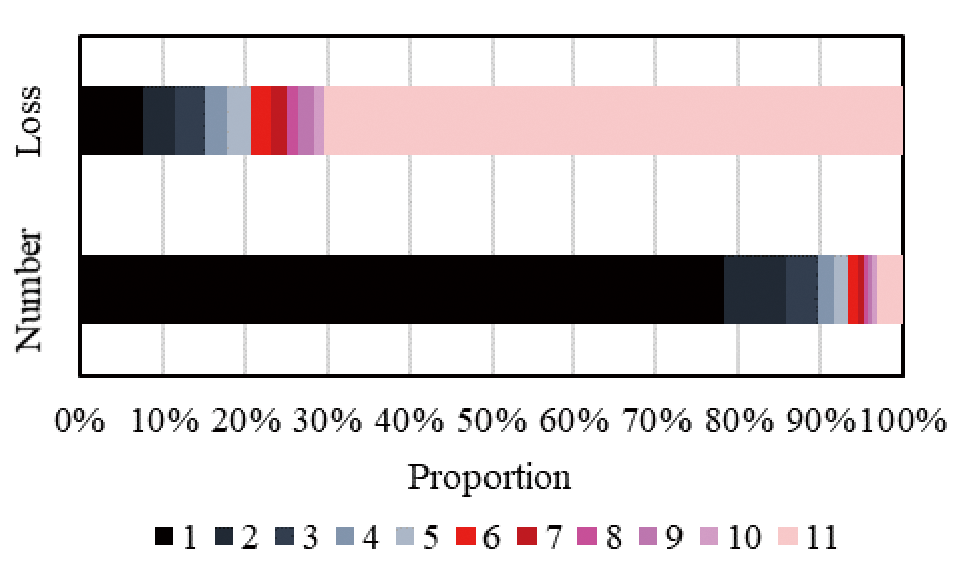

Supplement: S1 Fig — (TIF) [file pone.0190103.s001.tif]

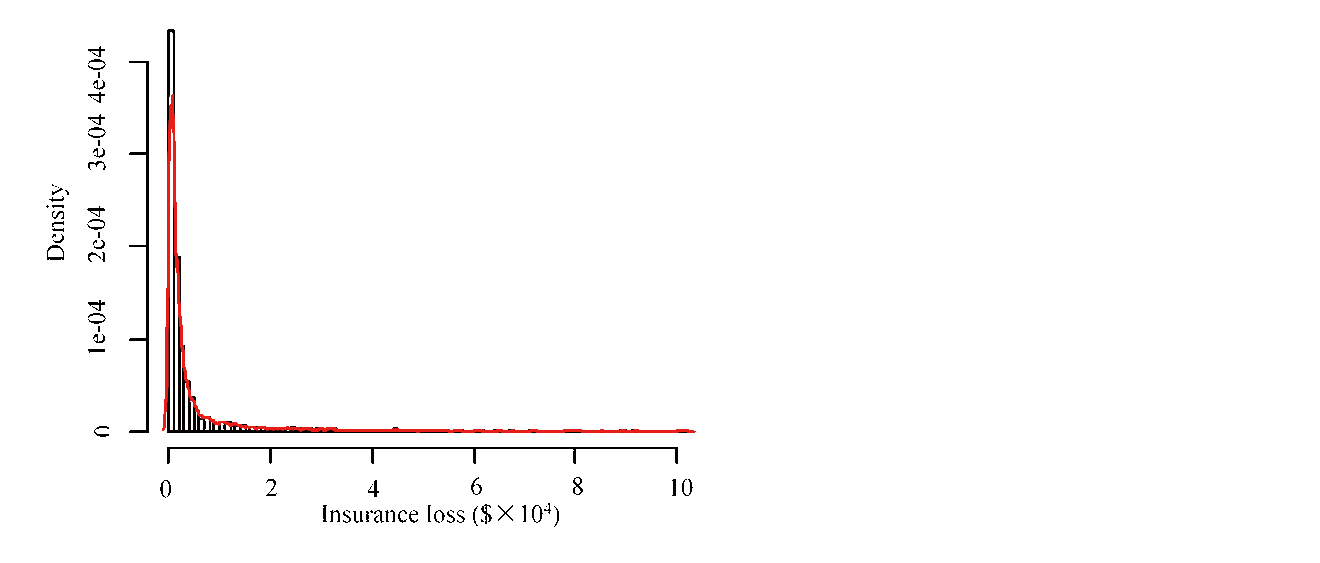

Supplement: S2 Fig — (TIF) [file pone.0190103.s002.tif]

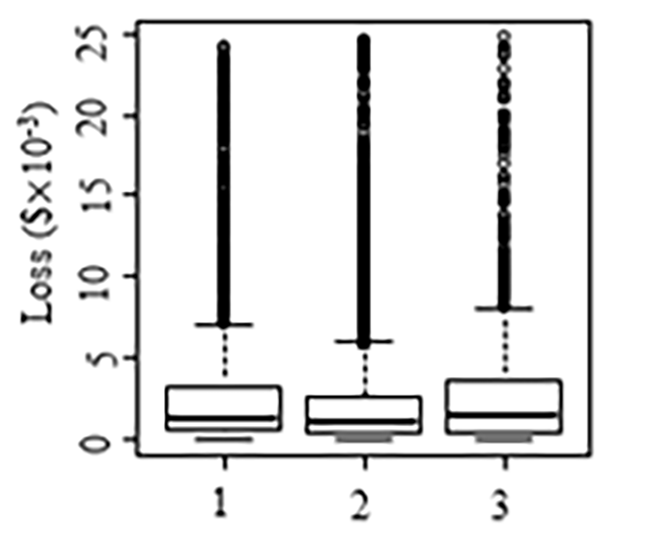

Supplement: S3 Fig — (TIF) [file pone.0190103.s003.tif]

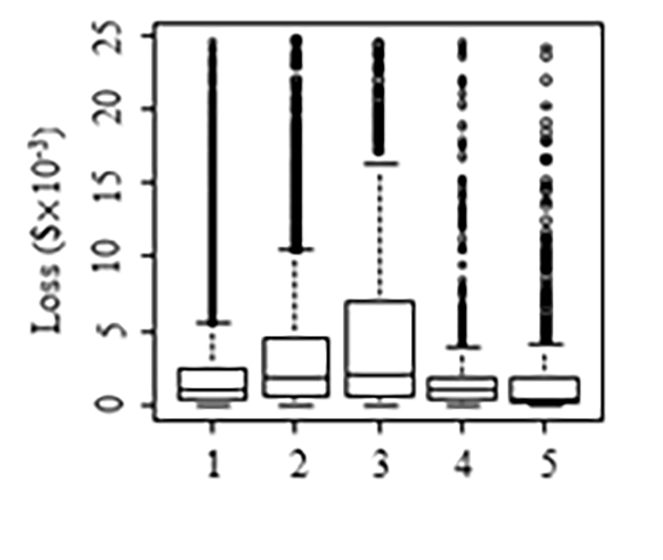

Supplement: S4 Fig — (TIF) [file pone.0190103.s004.tif]

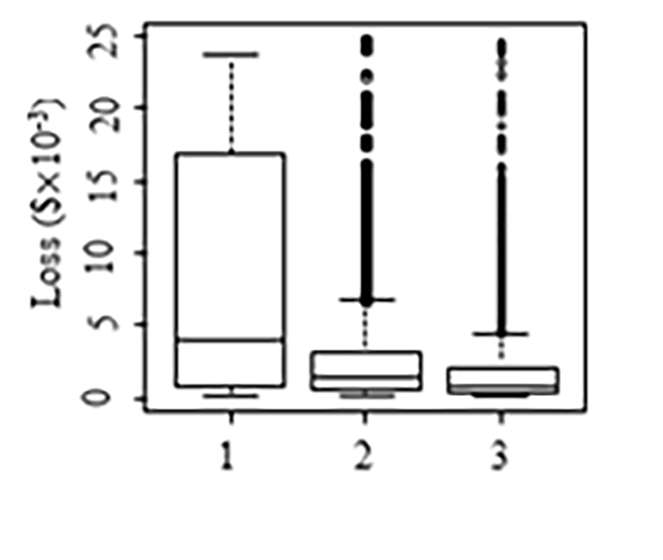

Supplement: S5 Fig — (TIF) [file pone.0190103.s005.tif]

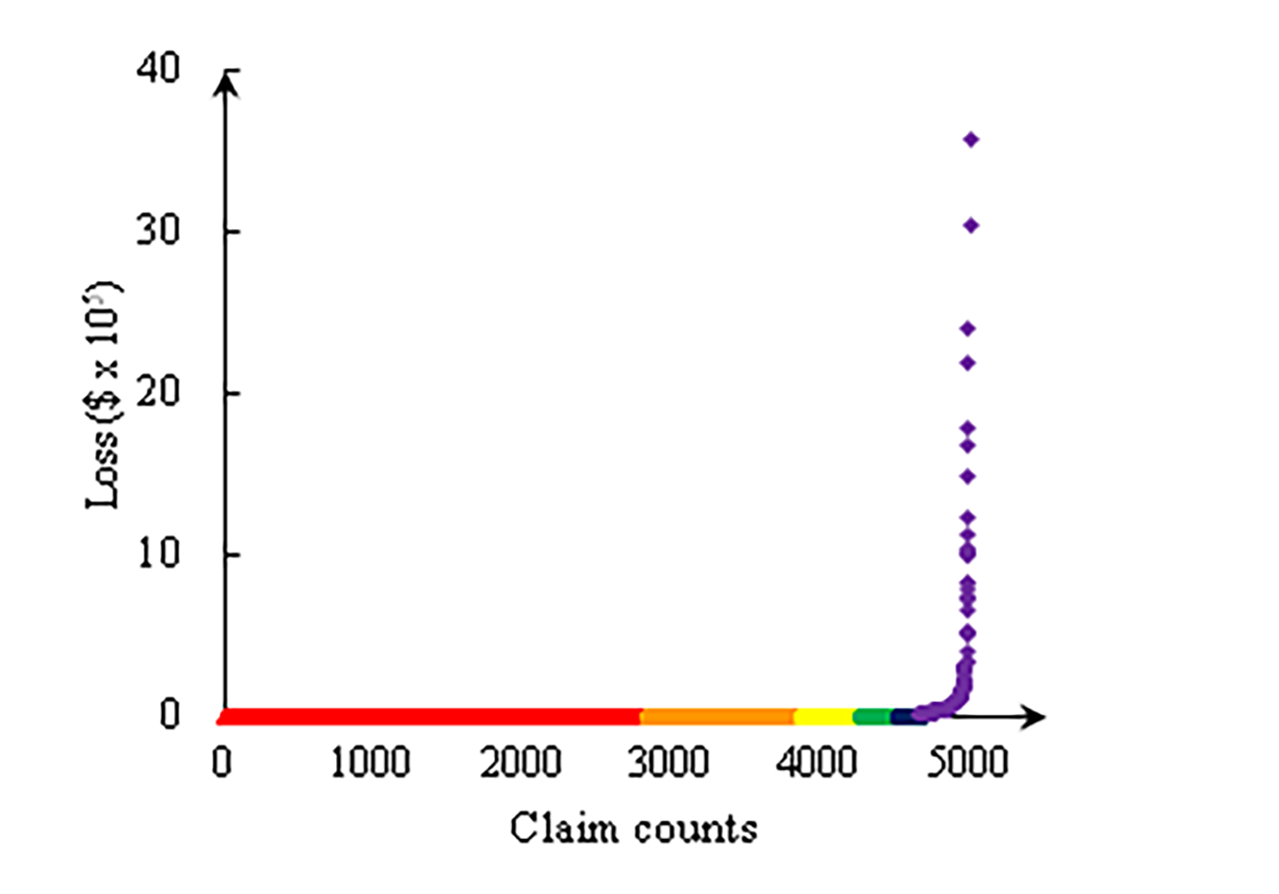

Supplement: S6 Fig — (TIF) [file pone.0190103.s006.tif]
